# The Third Annual Meeting of the European Virus Bioinformatics Center

**DOI:** 10.3390/v11050420

**Published:** 2019-05-05

**Authors:** Franziska Hufsky, Bashar Ibrahim, Sejal Modha, Martha R. J. Clokie, Stefanie Deinhardt-Emmer, Bas E. Dutilh, Samantha Lycett, Peter Simmonds, Volker Thiel, Aare Abroi, Evelien M. Adriaenssens, Marina Escalera-Zamudio, Jenna Nicole Kelly, Kevin Lamkiewicz, Lu Lu, Julian Susat, Thomas Sicheritz, David L. Robertson, Manja Marz

**Affiliations:** 1 European Virus Bioinformatics Center, 07743 Jena, Germany; bashar.ibrahim@uni-jena.de (B.I.); Stefanie.Deinhardt-Emmer@med.uni-jena.de (S.D.-E.); bedutilh@gmail.com (B.E.D.); volker.thiel@vetsuisse.unibe.ch (V.T.); Evelien.Adriaenssens@quadram.ac.uk (E.M.A.); kevin.lamkiewicz@uni-jena.de (K.L.); David.L.Robertson@glasgow.ac.uk (D.L.R.); 2 RNA Bioinformatics and High-Throughput Analysis, Friedrich Schiller University Jena, 07743 Jena, Germany; 3 Chair of Bioinformatics, Matthias-Schleiden-Institute, Friedrich Schiller University Jena, 07743 Jena, Germany; 4 MRC-University of Glasgow Centre for Virus Research, Glasgow G61 1QH, UK; s.modha.1@research.gla.ac.uk; 5 Department of Genetics and Genome Biology, University of Leicester, Leicester LE1 7RH, UK; mrjc1@leicester.ac.uk; 6 Institute of Medical Microbiology, Jena University Hospital, Am Klinikum 1, D-07747 Jena, Germany; 7 Section for Experimental Virology, Jena University Hospital, Hans-Knöll-Straße 2, D-07745 Jena, Germany; 8 Center for Sepsis Control and Care, Jena University Hospital, D-07747 Jena, Germany; 9 Theoretical Biology and Bioinformatics, Science4Life, Utrecht University, Padualaan 8, Utrecht 3584 CH, The Netherlands; 10 Centre for Molecular and Biomolecular Informatics, Radboud Institute for Molecular Life Sciences, Radboud University Medical Centre, Geert Grooteplein 26, Nijmegen 6525 GA, The Netherlands; 11 Infection & Immunity Division, Roslin Institute, University of Edinburgh, Midlothian EH25 9RG, UK; samantha.lycett@ed.ac.uk; 12 Nuffield Department of Medicine, University of Oxford, Peter Medawar Building, South Parks Road, Oxford OX1 3SY, UK; peter.simmonds@ndm.ox.ac.uk; 13 Institute of Virology and Immunology, 3012 Bern, Switzerland; jenna.kelly@vetsuisse.unibe.ch; 14 Department of Infectious Diseases and Pathobiology, Vetsuisse Facility, University of Bern, 3012 Bern, Switzerland; 15 University of Tartu, Institute of Technology, 50411 Tartu, Estonia; aare.abroi@ut.ee; 16 Quadram Institute Bioscience, Norwich Research Park, Norwich NR4 7UQ, UK; 17 Department of Zoology, University of Oxford, Parks Rd, Oxford OX1 3PS, UK; marina.escalerazamudio@zoo.ox.ac.uk; 18 Usher Institute of Population Health Sciences & Informatics, Ashworth Laboratories, Kings Buildings, University of Edinburgh, Charlotte Auerbach Road, Edinburgh EH9 3FL, UK; lu.lu@ed.ac.uk; 19 Institute of Clinical Molecular Biology, Kiel University, 24118 Kiel, Germany; jsusat@ikmb.uni-kiel.de; 20 Natural History Museum of Denmark, University of Copenhagen, DK-1123 Copenhagen, Denmark; thomassp@snm.ku.dk

**Keywords:** virology, virus bioinformatics, software, systems virology, metagenomics, virome, viral taxonomy, virus classification, genome evolution, bacteriophage, virosphere

## Abstract

The Third Annual Meeting of the European Virus Bioinformatics Center (EVBC) took place in Glasgow, United Kingdom, 28–29 March 2019. Virus bioinformatics has become central to virology research, and advances in bioinformatics have led to improved approaches to investigate viral infections and outbreaks, being successfully used to detect, control, and treat infections of humans and animals. This active field of research has attracted approximately 110 experts in virology and bioinformatics/computational biology from Europe and other parts of the world to attend the two-day meeting in Glasgow to increase scientific exchange between laboratory- and computer-based researchers. The meeting was held at the McIntyre Building of the University of Glasgow; a perfect location, as it was originally built to be a place for “rubbing your brains with those of other people”, as Rector Stanley Baldwin described it. The goal of the meeting was to provide a meaningful and interactive scientific environment to promote discussion and collaboration and to inspire and suggest new research directions and questions. The meeting featured eight invited and twelve contributed talks, on the four main topics: (1) systems virology, (2) virus-host interactions and the virome, (3) virus classification and evolution and (4) epidemiology, surveillance and evolution. Further, the meeting featured 34 oral poster presentations, all of which focused on specific areas of virus bioinformatics. This report summarizes the main research findings and highlights presented at the meeting.

## 1. Introduction

The European Virus Bioinformatics Center (EVBC) was conceived of in 2017 to bring together experts in virology and virus bioinformatics in Europe [1,2]. EVBC’s member numbers have increased steadily since then with currently 151 members from 78 research institutions distributed over 26 countries across Europe and internationally. This spring, the Annual Meeting of the EVBC was held for the third time (Table 1). The Third Annual Meeting of the EVBC attracted experts at all career stages to attend the two-day meeting in Glasgow in an inspiring and interactive scientific environment to promote discussion, exchange of ideas and collaboration and to inspire and suggest new research directions and opportunities.

## 2. Sessions and Oral Presentations

During the two-day conference, about 110 participants from 20 countries contributed in productive discussion on the four topics: (1) systems virology, (2) virus-host interactions and the virome, (3) virus classification and evolution and (4) epidemiology, surveillance and evolution. A number of high quality presentations were given by leading virologists and junior scientists. In addition to the eight invited speakers, we had twelve talks selected from the contributed submissions (see http://evbc.uni-jena.de/events/3rd-evbc-meeting). It was clear that the distinction between laboratory and computer researchers is often blurred. That collaborating teams of individuals with different skill sets are often a road to success, while individuals working alone can still make massive contributions. Data-driven research is now mainstream, and the scale and complexity of datasets is ever increasing. Discussions highlighted how virology, like all of biology, is now a data science, exploiting methods from dimensionality reduction of large datasets to data visualisation. We took from this that virus bioinformatics is evolving and succeeding as an area of research in its own right at the interface of virology and computer science and that there are many ways to be a successful researcher.

### 2.1. Systems Virology

This session was chaired by Philippe Le Mercier (University of Geneva Medical School, Switzerland), board member of the EVBC. Two speakers have been invited on this topic. Volker Thiel (University of Bern, Switzerland), board member of the EVBC, presented about host proteins composing the microenvironment of coronavirus replicase complexes. EVBC member Stefanie Deinhardt-Emmer (Jena University Hospital, Germany) presented about co-infection between *Staphylococcus aureus* and influenza virus. From the submitted abstracts, we selected talks by Jenna Nicole Kelly (University of Bern, Switzerland) on single-cell analysis of influenza virus infection, Florian Erhard (University of Würzburg, Germany) on tools revealing core features of CMV-induced regulation in single cells and Daniel Blanco Melo (Icahn School of Medicine at Mount Sinai, New York, USA) on in-depth transcriptomic analysis in influenza A virus infection. Studying virus infections at the molecular level is as complex as studying the host systems they infect.

#### 2.1.1. Determination of Host Proteins Composing the Microenvironment of Coronavirus Replicase Complexes, by Volker Thiel

**Figure 1:**
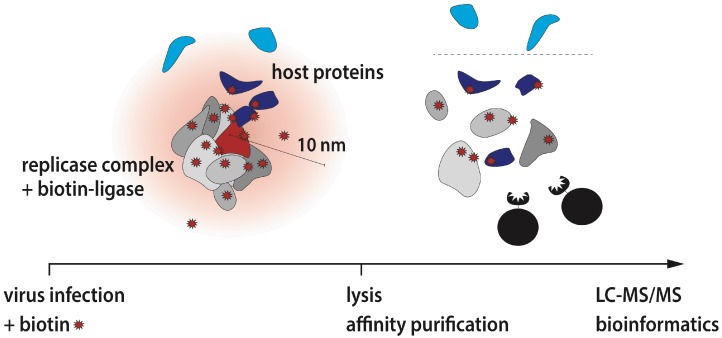
Illustration of the experimental design to determine the microenvironment of coronavirus Replicase Complexes (RCs) (adapted from V’kovski et al. [4]).

Coronaviruses are positive-sense RNA viruses that infect a variety of mammalian and avian species and are mainly associated with respiratory and enteric diseases. In humans, there are four coronaviruses known to cause rather mild respiratory symptoms; however, the appearance of zoonotic viruses, such as the Severe Acute Respiratory Syndrome (SARS) and Middle East Respiratory Syndrome (MERS) coronaviruses, exemplified that coronaviruses can also cause severe and lethal diseases in humans. Within their target cells, coronaviruses replicate their RNA genome at host-derived membranes in the host cell cytoplasm. The Replicase Complex (RC) that is synthesizing the viral RNA is encoded on the genomic RNA and comprises a set of 15–16 non-structural proteins (nsps). Besides canonical functions associated with RNA synthesis, such as RNA-dependent RNA polymerase, helicase and methyltransferases, a wealth of additional enzymatic activities, such as endoribonuclease, ADP-ribosylation and de-ubiquitination, are included within the coronaviral RC, suggesting that various virus–host interactions are taking place at the site of viral RNA synthesis. However, our knowledge about host factors at the interface between the RC and the host cell cytoplasm is rudimentary. To identify the composition of the viral RC and adjacent host cell proteins composing the RC-microenvironment, we engineered a biotin ligase into a coronaviral RC. This allowed us to biotinylate, affinity-purify and identify specifically all viral components constituting the coronavirus RC and host cell proteins that are in close proximity (Figure 1). Amongst the >500 host proteins constituting the RC-microenvironment, we identified numerous proteins associated with vesicular trafficking pathways, ubiquitin-dependent and autophagy-related processes and translation initiation. Notably, following the detection of translation initiation factors at the RC, we were able to visualize and demonstrate active translation proximal to the site of viral RNA synthesis of several coronaviruses. Collectively, our work established a spatial link between viral RNA synthesis and diverse host factors of unprecedented breadth. Many of the coronavirus RC-proximal host proteins and pathways have also documented roles in the life cycle of other positive-stranded RNA viruses, suggesting considerable commonalities and conserved virus-host interactions at the RCs of a broad range of RNA viruses. Our data may thus serve as a paradigm for other RNA viruses and provide a starting point for a comprehensive analysis of critical virus-host interactions that represent targets for therapeutic intervention [4].

#### 2.1.2. Co-Infection between *Staphylococcus aureus* and Influenza Virus Reduces Endothelial Barrier Function, by Stefanie Deinhardt-Emmer

Pneumonia is the most serious inflammatory disease of the respiratory tract and also the most common infectious disease. The classification of pneumonia into Hospital-Acquired Pneumonia (HAP), Community-Acquired Pneumonia (CAP) and Ventilated-Acquired Pneumonia (VAP) indicates the source of disease by a wide variety of microorganisms including bacteria, viruses and fungi. Respiratory tract infections and in particular pneumonia represent the most common cause of sepsis [5]. Long-time associated with bacterial infection, sepsis definition became more in focus as a multifaceted host response to an infecting pathogen, which leads to organ failure [6]. However, Influenza Virus (IV) as a pneumotropic virus can lead to lung failure and systemic host reaction with subsequent multiple organ failure. IV circulates worldwide and causes highly contagious respiratory diseases characterized by mild to severe symptoms. The seasonal IV-associated bronchopneumonia is one of these infectious diseases with the highest population-based mortality rates [7]. Besides virulence factors, the sudden increase of pathogenicity is the most striking problem of influenza accompanied by bacterial co-infection. In a single-centre study conducted at the Jena University Hospital during the winter season 2017/2018, we detected 1197 influenza-virus-positive samples and 89 *S. aureus*-positive respiratory specimens. However, the diagnosis of a co-infection was significantly lower with 17 samples. Interestingly, the mortality rate increased dramatically from single infection (approximately 20%) to co-infection (approximately 80%). Even larger studies indicating similarly dates and also the Spanish flu of 1918 showed that co-infection results in high mortality rates [8]. While the pathogen–host interaction-induced severe dysregulations of the immune response is under investigation in many studies, the regulatory effects between the different pathogens and the subsequent impact on the host are barely understood. In a multifactorial process, a wide range of pathogen factors and pathogen-regulated signalling events are involved in co-pathogenesis. This process is associated with elevated host-response, changed repair-processes, and modifications in the cellular immune response [9]. It is shown that primary IV-infection inhibits the apoptosis mechanism and the following infection with *S. aureus* inhibits IV-induced apoptosis by procaspase-8 activation [10]. Various models are available for studying the mechanisms of the viral–bacterial interference. However, the use of murine models is adversely regarded because of obvious discrepancies between men and mice despite the attempts of humanized murine models to fill the gaps. New methods enable investigations with cost-saving and efficient cell culture models as an excellent supplement to animal experiments. Organ-on-a-chip technology allows species-specific investigations for different cell types and also immune cells. Using this method, viral-bacterial interference can be investigated in a human-specific manner.

#### 2.1.3. Single Cell Analysis of iNfluenza Virus Infection in Its Natural Target Cells Reveals Cell Type-Specific Host Responses and Disparate Viral Burden, by Jenna Nicole Kelly

**Figure 2:**
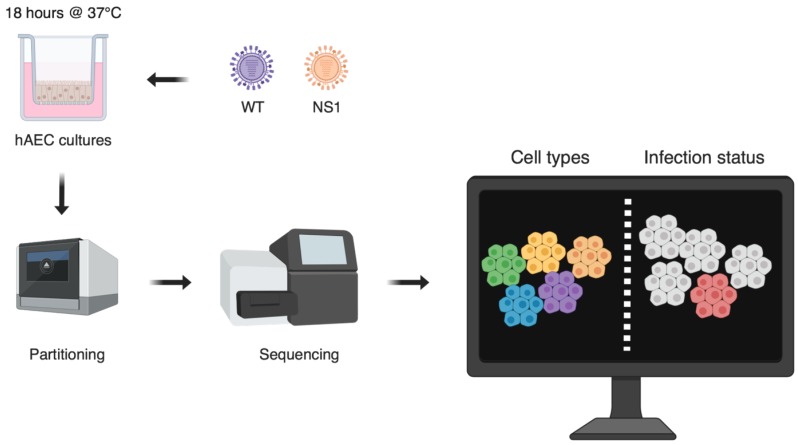
Sequencing and annotation workflow for single influenza-infected cells in the human respiratory epithelium. hAEC, human Airway Epithelial Cells.

The human respiratory epithelium is a pseudostratified epithelium that constitutes the first line of defence against invading respiratory pathogens, including influenza viruses. Although several studies have now shown that both viral transcript production and the innate immune response to infection vary widely among single influenza-infected cells, the cause of this extreme heterogeneity remains unclear [11,12]. More specifically, it remains unknown how key innate immune components are distributed among the different cell populations found in the respiratory epithelium and how the latter may influence the host response to infection. To determine the distribution of these innate immune components and to examine how specific cell types respond to influenza infection, we used single-cell RNA sequencing to acquire transcriptomes from primary human Airway Epithelial Cells (hAEC) infected with Influenza A Virus (IAV) (Figure 2) [13]. A low MOI was used to infect hAECs with either Wild-Type (WT) pandemic IAV or an NS1mutated form of the virus (NS1R38A) that impairs its ability to counteract Interferon (IFN) and produces an amplified innate immune response. We then annotated both host and viral transcriptomes of more than 19,000 single cells across the five major hAEC cell types for mock, WT, and NS1R38A conditions. We observed a large heterogeneity in viral burden; however, in contrast to what was found in previous studies, no absence of viral genes was detected. Interestingly, in both WT- and NS1R38A-infected cultures, there was a significant decrease in the fraction of ciliated and goblet cells compared to mock hAECs. We also identified a number of cell-type-specific innate immune responses, including the expression of type I and III IFNs in all major cell types. Collectively, our results represent the first comprehensive report on how individual cells contribute to the antiviral response during IAV infection in the context of the human respiratory epithelium.

### 2.2. Virus–Host Interactions and the Virome

This session was chaired by David Robertson (MRC-University of Glasgow Centre for Virus Research, United Kingdom), the local organizer of the meeting. Bacteriophages, the viruses of bacteria, are an important and usually neglected component of microbiome studies. Two speakers have been invited on this topic. Martha Clokie (University of Leicester, United Kingdom) presented about the roles of phages in impacting infectious diseases in human microbiomes. EVBC member Bas Dutilh (Utrecht University, Netherlands) presented about global phylogeography and the ancient evolution of the widespread human gut virus crAssphage. From the submitted abstracts, we selected talks by Katherine Brown (University of Cambridge, United Kingdom) on viral transcripts in RNA-seq datasets from bees, mites, and ants, Evelien Adriaenssens (Quadram Institute Bioscience, Norwich, United Kingdom) on genome-resolved metaviromics for the detection of pathogenic viruses in the environment and Josquin Daron (Centre National de la Recherche Scientifique, Montpellier, France) on codon usage preference similarity among human-infecting viruses and their hosts.

#### 2.2.1. Roles of Phages in Impacting Infectious Diseases in Human Microbiomes, by R. J. Martha Clokie

Most of the roles of phages in human health and disease are yet to be unravelled. However, phages in all environments including the human microbiome are increasingly acknowledged to be the puppeteers of their bacterial hosts, shaping their structure and evolution and physiology. Phages associated with bacterial pathogens have multiple, often complex interactions with their bacterial hosts, forcing them to interact differently with other bacterial and human cells. Besides being the ultimate bacterial killers, phages can change bacterial surfaces to prevent recognition by the human immune system. In cystic fibrosis, they can allow their hosts to cope with anaerobic conditions found in mucus-laden lungs, and in many bacteria, they encode potent toxins [14]. There is indeed a plethora of unknown phage-mediated bacterial phenotypes that could be critical for our understanding of disease. Their ability to be developed as targeted removers of pathogenic bacteria is likely to be critical to solving the antimicrobial resistance crisis.

A major limitation for our ability to develop therapeutic phages and also understand fully the ways that phages impact bacteria is that the vast majority of phage gene functions are hypothetical or unknown. In bacterial genomes, there are around 25% unknown genes, or genes that have no known ascribed function, but in phage genomes, only around 25% of the genes are generally known! Thus, when trying to establish how phages specifically interact with their hosts, there is large number of genes of which we need to try and make sense.

**Figure 3:**
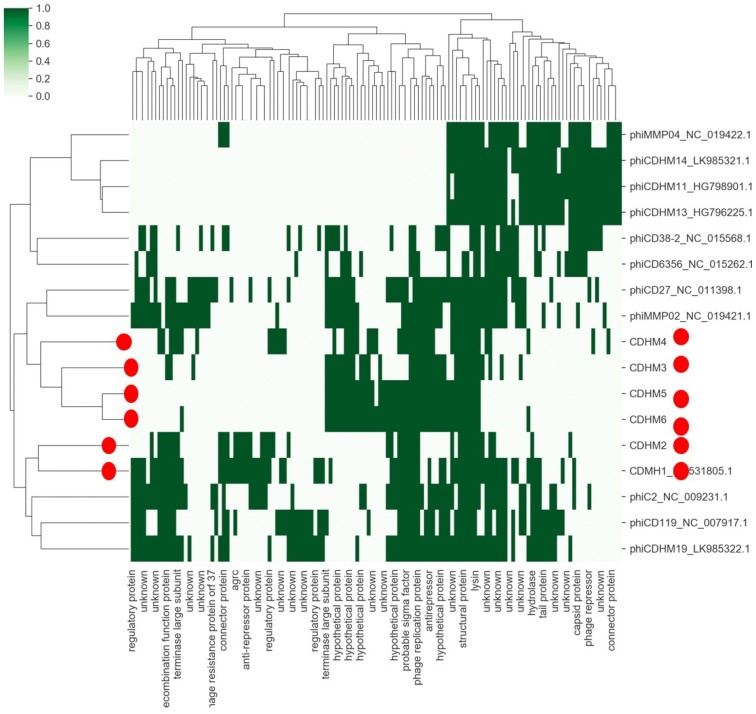
Set of *Clostridium difficile* phages on the vertical axis, which includes six well-characterised myoviruses from Martha Clokies’ laboratory (red dots). The genes commonly identified in *C. difficile* phages are shown on the horizontal axis and homologous genes represented by a green line. It is clear that these phages do not share a large common gene set.

To illustrate the diversity within one specific phage set, Martha Clokie presented the work from her lab on phages that infect the gut pathogen *Clostridium difficile* [15,16,17]. They have identified sets of phages that target clinically-relevant and prevalent strains. Despite the most effective phage set being isolated from one geographical location, they are strikingly variable (Figure 3) with very few identifiable genes in common.

Martha Clokie’s group is currently in the process of creating and examining genetic mutants to identify phenotypes and conducting structural work on novel proteins, for example to identify tail fibres. However, this work is time consuming and technically demanding. Choosing which genes to focus on is key, as downstream work is key to unravelling critical phenotypes. Martha Clokie presented data on the efficacy of this phage set to treat disease along with a framework for their ongoing work to use different machine learning approaches to examine the genomes of these phages and their associated bacteria robustly in order to identify hard-to-identify features, for example shared and unique genes of interest. These approaches will direct work to unravel the mechanics of phage efficacy for virulent phages and modes of action for lysogens.

#### 2.2.2. Global Phylogeography and Ancient Evolution of the Widespread Human Gut Virus crAssphage, by Bas E. Dutilh

While viruses are vastly abundant and ubiquitous throughout the biosphere, they have remained a relatively unexplored superkingdom of life. Early findings of genomic mosaicism [18] and enhanced mutation rates of especially RNA viruses [19] have led to the conception of viruses as genomically highly variable entities. This was further supported as metagenomics unveiled the extent of genetic diversity of viruses, initially in marine water and human faeces [20], and in many different biomes since. Images of an unparalleled diversity that is dominated by unknown sequences has been the common theme of viral metagenomic explorations. However, while the virosphere is undoubtedly diverse, ubiquitous viruses are increasingly being discovered by metagenomic analysis of globally-distributed, ecologically-stable ecosystems, including once again the global oceans [21,22] and the human gut [23,24,25].

Moreover, the genome sequence in individual viral lineages may be more conserved than could previously be recognized. Recently, large-scale comparisons of gene order in the genome sequences of dsDNA bacteriophages revealed a surprisingly conserved genomic structure [26,27]. A possible mechanism at play is the genomic encoding of different transcriptional regions with promoters that govern the expression of early, middle and late specific genes, such as known from the well-studied case of the T4 bacteriophage [28]. Together, these findings suggest a highly-optimized genomic encoding of gene expression regulation that is consistent across globally-diverse viral populations.

While the conservation of genomic architecture between distantly-related bacteriophages as outlined above is a striking observation, many open questions remain. For example, it remains unclear to what extent the observations of conserved genomic architecture described above reflect a biased sampling, for example of temperate, dsDNA and/or tailed bacteriophages that have been observed to dominate, e.g., marine systems [29]. Indeed, the modes of genome evolution differ for viruses with different lifestyles [30]. Nevertheless, viruses have vast global population sizes that result in highly-efficient evolutionary selection pressures and optimized genomes. Moreover, viruses and their cellular hosts have been co-evolving for billions of years, allowing ample time for optimization of their genome structures.

Viral mutation rates (including recombination rates) have remained difficult to quantify due to a lack of evolutionary calibration points. For example, on a short time scale of thirty years, a constant recombination rate of five events per year has been observed for Siphoviridae bacteriophages [31], but when longer timespans are assessed, mutation rate estimates may drop dramatically by orders of magnitude [32]. One way of obtaining ancient calibration points in viral evolution in the absence of fossil data is by exploiting their association to hosts. One of the most conserved constituents of the human gut virome is the widespread and abundant bacteriophage crAssphage [23]. Recently, near-complete genome sequences of crAss-like viruses were detected in faecal samples of a range of wild non-human primates living on different continents, including Old-World monkeys, New-World monkeys and apes [33]. Strikingly, these genomes revealed a strong collinearity with human-associated crAss-like viruses, suggesting that the association of crAss-like viruses with the primate gut biome may be millions of years old. Moreover, these findings open the door to investigations into viral mutation rates at long time-scales, once again illustrating how viral metagenomics opens up a treasure trove for virus discovery [34], as well as evolutionary analyses of these smallest and most abundant biological entities on Earth.

#### 2.2.3. Genome-Resolved Metaviromics for the Detection of Pathogenic Viruses in the Environment: Will Eating Shellfish Make You Ill?, by Evelien M. Adriaenssens

Viromics or viral metagenomics has been proposed as an alternative method to qPCR-based approaches for the detection of pathogenic viruses linked to food- and water-borne illness in the aquatic environment [35,36]. The main advantage is that viral communities can be investigated without prior knowledge of the genome sequences or genotypes of the viruses present in the sample. There are, however, several drawbacks associated with viromics, such as laboratory and computational costs, scalability and the issue of viral dark matter in which sequence data are classified as “unknown”. In her presentation, Evelien Adriaenssens focused on the latter aspect and showed that reconstruction of Uncultivated Virus Genomes (UViGs) [37] and classification into families reduced the fraction of completely unknown sequences, particularly for RNA viruses. Using read mapping approaches followed by visualisation and analysis with Anvi’o [38], she showed that they can identify pathogenic virus genomes present in the Conwy River catchment area, mainly found in wastewater [39], and showed changing abundance patterns between sample sites and types. Using species-level clustering and differential read mapping, comparative genomics and phylogenetics, she could gradually descend from the bigger picture of viral diversity to strain-level resolution, identifying the genotype of potentially pathogenic viruses. This workflow is ideally suited to find new pathogenic viral species and identify markers for wastewater contamination of the environment.

Evelien M. Adriaenssens was funded by the Biotechnology and Biological Sciences Research Council (BBSRC) under the BBSRC Institute Strategic Programme Gut Microbes and Health BB/R012490/1.

### 2.3. Virus Classification and Evolution

This session was chaired by Darren Obbard (University of Edinburgh, United Kingdom). Two speakers have been invited on this topic. Peter Simmonds (University of Oxford, United Kingdom) presented about classification of viruses in metagenomic datasets. Unfortunately, Olga Kalinina (Max Planck Institute for Informatics, Saarbrücken, Germany) was unable to make it to the meeting. Instead, Manja Marz (Friedrich Schiller University Jena, Germany), Managing director of the EVBC, presented about machine learning applied to virus data. From the submitted abstracts, we selected talks by Julian Susat (Institute of Clinical Molecular Biology, Kiel, Germany) on the detection of viruses in ancient human remains, Aare Abroi (University of Tartu, Estonia) on the relation between virosphere and biosphere, and Kevin Lamkiewicz (Friedrich Schiller University Jena, Germany) on RNA secondary structures in whole genome alignments of viruses. Based on this submission, Kevin was competitively awarded the PhD travel award.

#### 2.3.1. The Classification of Viruses in Metagenomic Datasets—Where Do You Draw the Line?, by Peter Simmonds

Methodological advances, such as High-Throughput Sequencing (HTS), and new capabilities to recover and assemble genome sequences has unearthed vast numbers of previously-undescribed viruses from environmental, human clinical, veterinary and plant samples. How such viruses can be incorporated into the current virus taxonomy is a major challenge, especially at the family and species levels, which have been historically based largely on descriptive taxon definitions of phenotypic properties that “sequence-only” viruses often lack. These assignments typically encapsulate descriptions of replication strategies, virion structure, and clinical and epidemiological features, such as host range, geographical distribution and disease outcomes. If “sequence-only” viruses are to be formally placed into the classification maintained by the International Committee on the Taxonomy of Viruses (ICTV) as recently proposed [40], then their assignments will have to be based largely or entirely on metrics of genetic relatedness and any other features that might be inferred from their genome sequences. However, there are no published guidelines in the ICTV code on how similar or how divergent viruses must be in order to be considered as new species or new families (https://talk.ictvonline.org/information/w/ictv-information/383/ictv-code).

**Figure 4:**
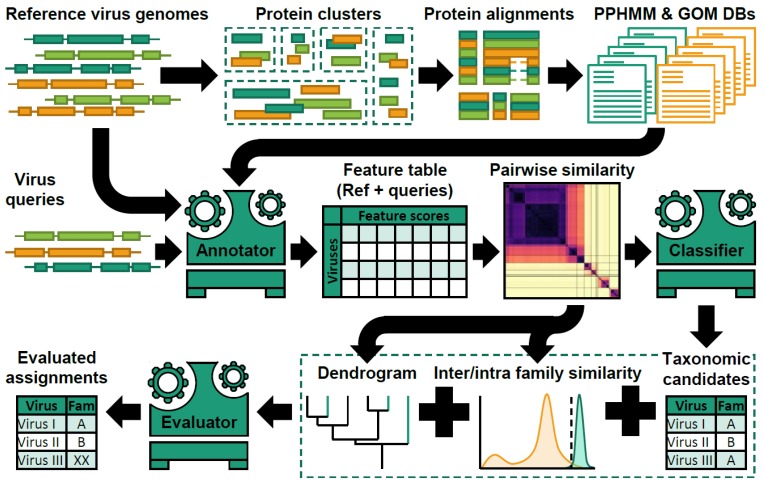
Overview of virus taxonomy prediction by “Genome Relationships Applied to Virus Taxonomy” (GRAViTy). A simplified diagram of the steps used to construct profile tables from sequences of viruses with assigned taxonomic status (reference virus genomes). It further illustrates the steps to classify viruses of of undetermined taxonomic relationships. The method is based on extraction of protein sequences from reference virus genomes and their clustering using pairwise BLASTp bit scores. Sequences in each cluster are then aligned and turned into a Protein Profile Hidden Markov Model (PPHMM). Reference genomes are subsequently scanned against the database of PPHMMs to determine the locations of their genes, and Genomic Organisation Models (GOMs) for each virus family are constructed. These models form the core of the genome annotator (Annotator), which is used to annotate query sequences with information on the presence of genes and the degree of similarity of their genomic organisation to reference virus sequences. From this, genome relationships can be extracted by computation of various genetic distance metrics, including composite generalised Jaccard similarity, which forms the basis for heat maps and dendrograms that depict the relationships of query sequences to the dataset of classified viruses (Classifier) and recommendations for their taxonomic assignments (Evaluator).

Peter Simmonds described their investigations of the extent to which the existing virus taxonomy could be reproduced by the recoverable genetic relationships between sequences of viruses currently classified by the ICTV. Comparisons of viruses were based on extraction of protein coding gene signatures and genome organisational features from virus sequences and using these to construct a metric of genetic relatedness through computation of Composite Generalised Jaccard (CGJ) distances between each pair of viruses [41]. For eukaryotic viruses, there was large-scale consistency between such genetic relationships and their current family- and genus-level taxonomic assignments, irrespective of genome configurations and genome sizes. The analysis pipeline, “Genome Relationships Applied to Virus Taxonomy” (GRAViTy), diagrammatically summarised in Figure 4, predicted family membership of eukaryotic viruses with close to 100% accuracy and specificity; this method should therefore enable the vast collection of metagenomic sequences to be classified in a manner consistent with the current ICTV taxonomy. Preliminary analysis of such datasets revealed that over one half (460/921) of (near)-complete genome sequences from recently-generated eukaryotic virus datasets could be assigned to 127 novel family-level groupings, more than double the number of eukaryotic virus families in the ICTV taxonomy.

The taxonomy of the 20 currently-classified prokaryotic virus families differs substantially [42]. Members of three families in particular (*Podoviridae*, *Siphoviridae* and *Myoviridae*) were far more divergent from each other than observed within eukaryotic and archaeal virus families. Applying a CGJ distance threshold of 0.8, prokaryotic viruses form over 100 groupings equivalent to eukaryotic virus families. The use of a common benchmark with which to compare taxonomies of eukaryotic and prokaryotic viruses supports ongoing efforts by the ICTV to revise thoroughly the phage taxonomy so that assignment criteria are consistent across all virus groups. Developing a consistent classification of viruses in which assignments at family and other taxonomic levels extending the current framework, but which will be underpinned both by metrics of genomic relatedness, is essential for future, evidence-based classification of metagenomic viruses.

#### 2.3.2. Detecting Viruses in Ancient Human Remains, by Julian Susat

**Figure 5:**
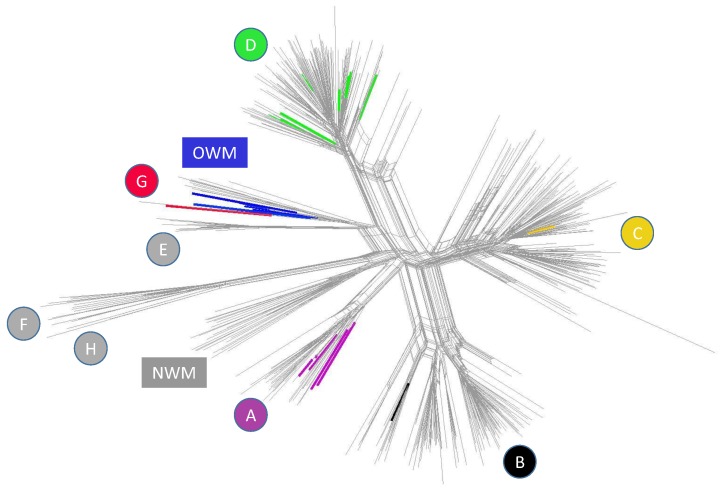
Network of 493 modern genomes, 15 published ancient strains and 12 newly-discovered ancient strains. Single letters indicate HBV genotypes (A–H); coloured strains are of ancient origin; OWM = Old-World Monkey HBV strains, NWM = New-World Monkey HBV strains. D: five new ancient strains, six ancient strains [43,44,45]; C: one ancient strain [46]; B: one ancient strain [45]; A: two new ancient strains, three ancient strains [45]; G: one new ancient strain; OWM: three new ancient strains, four ancient strains [44,45].

The field of ancient DNA covers a wide range of research topics, spanning from human evolution, megafauna to pathogen evolution. Despite the recent advantages in ancient DNA techniques and modern metagenomic screening tools, the identification of authentic viral sequences from ancient material is still challenging. The materials that are mainly used in ancient DNA research, teeth and petrous bones, already limit the number of detectable viruses by their nature. Only viruses that are present in the bloodstream can be detected. The fast evolution of viral pathogens and therefore the comparability to modern variability in viruses makes it even more difficult to identify their ancestors reliably. The highly-fragmented and degraded nature of ancient genetic material and the high risk of modern contamination are causing further problems in the analysis. For the detection of viruses, a wide variety of software utilizing different approaches like HMMs, dedicated marker genes and complete genome references are available to screen these ancient samples for the presence of pathogens. Each of these approaches has its own characteristic strengths and weaknesses. In a competitive alignment approach using all complete virus genomes as a reference, we were able to detect three Hepatitis-B Viruses (HBV) during our regular screening. All three samples originated in Germany and dated to the mediaeval times (1000 BP) and the Neolithic (5000 and 7000 BP). After sequencing and competitive mapping against 16 HBV references, complete HBV genomes could be recovered from all three samples. This resulted in the oldest human pathogenic viral genome that is known up to know. Phylogenetic analysis revealed that the medieval strain was genotype D and surprisingly conserved. The ancient Neolithic strains were closer together than to any other modern and closest to strains from Old-World monkeys. These findings might suggest reciprocal cross-species transmission between human and ape. Furthermore, we could show that the genomic structure of ancient strains closely resembles the structure of modern HBV strains. Since publishing these results, we and others detected more HBV-positive samples, supporting the notion that viruses will become more important for the aDNAcommunity (Figure 5). The new HBV genomes we reconstructed support our earlier findings. A bigger number of HBV cases spanning over longer time frames opens the door for reliable diachronic analysis and maybe even epidemiological analysis. Besides the recent findings of ancient viruses (e.g., Parvovirus), an open question still remains how we could detect and reconstruct extinct or highly-altered virus genomes. Bioinformatic protocols for the detection of unknown viral protein families based on long sequencing reads and high coverage data are published and available, but due to the above-described nature of aDNA, applying these methods is not straightforward, and strong optimization needs to be carried out. Still, these HBV and other findings have opened a new door within the aDNA community and blazed a trail for upcoming viral ancient DNA studies. This work was done by a team composed of Ben Krause-Kyora, Julian Susat, Felix M. Key, Denise Kühnert, Alexander Immel, Alexander Herbig, Almut Nebel and Johannes Krause.

#### 2.3.3. Virosphere and Biosphere—How Related They Are? A Protein (Domain) Based View, by Aare Abroi

**Figure 6:**
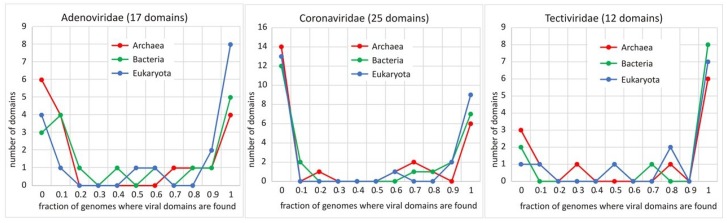
Distribution of the protein domains found in three viral families according to their occurrence in different superkingdoms. Protein domains as they are defined in SCOPat the superfamily level and the occurrence of these domains according to Superfamily assignment (www.supfam.org). For example, Coronaviridae encodes 13 protein domains not found in eukaryotic genomes and nine domains found in more than 90% of eukaryotic genomes.

Viruses are not always pathogens, and they are also an important and inseparable part of the biosphere and should be studied as such. Unfortunately, the wider functional and evolutionary role of viruses in the biosphere is not yet widely accepted in most disciplines, a good exception being marine biology/ecology, where viruses are already accepted as important players. How the virosphere is related to the rest of the biosphere can be examined in several different ways. One of these ways is a protein domain-based view. We analysed how virosphere protein domain occurrence is related to the occurrence of protein domains in all (sequenced) organisms (we called the last the phylogenomic space of protein domains). This is based on the distribution of protein domains in viruses and in organisms (by superkingdom), i.e., which protein domains are found in viruses (or a specific set of viruses) and to what extent and where these domains are found elsewhere in organisms. In our analysis, we used predefined protein domain databases Pfam, Superfamily and Gene3D. Domains found in the virosphere can be found in a different number of organisms, starting from a few organisms for some viral domains up to all organisms in the others. However, if we specify a narrower set of viruses (Baltimore class, viral family or host range), differences between viral taxons appear. Therefore, the heterogeneity of viruses is also very clearly expressed by where in the phylogenomic space the domains that are found in different viral taxons are located. A few examples are shown in Figure 6. An important conclusion from our analysis is the existence of virosphere-specific protein domains (domains not found in cellular organisms), even at the level of structural homology. Several evolutionary routes that may lead to virosphere specificity (absence in cellular organisms) will be discussed. Considering the new knowledge on virus-to-host gene transfers in eukaryotes during the last ten years, it is clear that the virosphere is a source of functional and structural novelties also for this superkingdom. A possible route for the genesis of novel domains in viruses (as well as in organisms) is double coding or overprinted genes. We have developed a web-tool cRegions (http://bioinfo.ut.ee/cRegions/), which helps to find potential double coding regions (and other embedded functional elements) in coding sequences [47,48]. Of course, there exist many domains that are shared by viruses and organisms. Beside others, virus-to-host gene transfer is one process leading to shared domains. A number of examples for this kind of transfer have been described; however, they are all based on sequence-to-sequence comparison. Taking into account the very fast evolution of viruses, the sequence similarity may fall below the confidential detection limit relatively fast. We applied structure-guided information to detect more ancestral virus-to-host transfers. Our data show that “as a proof of principle”, using protein structure-guided HMM models, it is possible to detect V2Htransfers not “visible”; with BLAST analysis.

#### 2.3.4. RNA Secondary Structures in Whole Genome Alignments of Viruses, by Kevin Lamkiewicz

RNA secondary structures are known to play important roles in viruses, and especially in RNA viruses, since they can initiate and facilitate transcription, translation and replication. Several studies indicate that structures are cis-acting regulators for transcription. However, only looking at local structures is not sufficient to capture all RNA–RNA interactions of one molecule. Long-Range Interactions (LRI) are described in a few RNA virus families [49], but are computationally intensive to predict. Further, studies show that a single nucleotide changing can disrupt the replication of a coronavirus completely [50]. Thus, a deep understanding of conserved RNA structures is necessary to develop anti-viral therapies.

In order to increase the confidence of predictions, Multiple Sequence Alignments (MSA) are needed, since they provide conservation information between viruses. Identifying conserved secondary structures in whole genomes of viruses is computationally challenging, as the whole genome has to be considered for possible structures and interactions.

**Figure 7:**
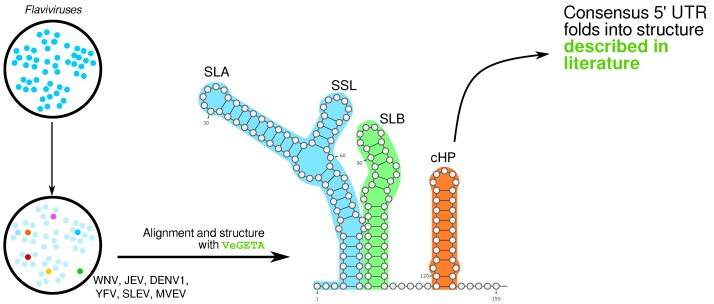
First results of our VeGETApipeline on an example input set consisting of flaviviruses. We were able to identify the West-Nile Virus (WNV), Dengue Virus 1 (DENV1), Japanese Encephalitis Virus (JEV), Yellow Fever Virus (YFW), Saint Louis Encephalitis Virus (SLEV) and Murray Valley Encephalitis Virus (MVEV) as representative viruses from downloaded virus genomes [53]. The resulting alignment calculated by VeGETA has structure annotations for the complete genomes, including 5’ UTR, coding regions and 3’ UTR. Here, we extracted the 5’ UTR from the alignment and visualized the annotated structure elements. These elements agree with the literature [54], as we were able to reconstruct the SLA, SLL, SLBand cHPelements accurately. The first two elements were recognized by the viral replication mechanism (NS5) [55]. The sequence embedded in the SLB structure is known to play a role in the genome circularization of flaviviruses [56], whereas the cHP facilitates the translation of the coding region by pausing the translation machinery and finding the correct starting triplet [57].

Here, we give an overview of the landscape of RNA secondary structures in viruses and provide a pipeline that generates whole genome alignments with structure annotation for downstream analyses. Our pipeline distinguishes itself from other tools by considering both the sequence and structure of input genomes for the final alignment. Therefore, for the first time, the generation of structure-annotated whole genome alignments for viruses enables sophisticated and comprehensive downstream analysis for RNA structures and RNA functions. This is achieved with an iterative combination of the sequence-based aligner MAFFT [51] and the structure-based aligner LocARNA [52]. For our example case, we were able to predict structures in the genus *Flavivirus* [53] that are consistent with described structures in the literature (Figure 7). Further, we predicted novel structural elements in coding regions of genomes.

### 2.4. Epidemiology, Surveillance and Evolution

This session was chaired by Edward Hutchinson (MRC-University of Glasgow Centre for Virus Research, United Kingdom). Two speakers have been invited on this topic. Samantha Lycett (University of Edinburgh, United Kingdom) presented about phylodynamics for tracking epidemic, endemic and evolving viral strains. Roman Biek (University of Glasgow, United Kingdom) presented about leveraging pathogen genomics to reveal and control the spread of rabies virus. From the submitted abstracts, we selected talks by Marina Escalera-Zamudio (University of Oxford, United Kingdom) on parallel evolution and the emergence of highly-pathogenic avian influenza A viruses, David Bauer (University of Oxford, United Kingdom) on the structure of the influenza A virus genome and Lu Lu (University of Edinburgh, United Kingdom) on the evolutionary origins of the epidemic potential among human RNA viruses.

#### 2.4.1. Phylodynamics for Tracking Epidemic, Endemic and Evolving Viral Strains, by Samantha Lycett

Infectious diseases caused by viral pathogens in animal and livestock populations can have important economic and health consequences globally. The ability to foresee where, in which host species and under what conditions outbreaks could occur is key to developing prevention and control strategies. Sequencing pathogens from infected animals has become much more affordable and widespread in recent years, especially during outbreaks and in endemic disease settings with targeted surveillance programmes. Consequently, there are growing collections of animal virus sequences from around the globe. In this talk, the use of viral sequence data together with phylodynamic methodologies for understanding the transmission patterns in animal populations was discussed, using Avian Influenza (AI), Foot-and-Mouth Disease (FMD) and Porcine Reproductive and Respiratory Syndrome (PRRS) as examples [58,59,60].

Since RNA viruses have fast mutation rates and variable sequences, transmission routes between places and host species can be inferred [59,60]. One approach is to group sequences from individual hosts into discrete locations and/or host species and consider these as discrete traits or subpopulations on time-resolved phylogenetic trees, with the goal to infer which group infected which. Alternatively, locations may be represented as continuous traits (latitude and longitude) in order to estimate spatial diffusion rates and routes.

Using avian influenza as an example of a widespread multi-species disease system, it was shown that wild birds (wild Anseriformes) were responsible for long-range transmissions of highly-pathogenic H5N8, by using a combination of discrete host traits and continuous spatial traits on time-resolved phylogenetic trees [58]. Furthermore the clade to which the H5N8 strains belong is unusual because unlike the highly-pathogenic H5N1 strains, they reassort frequently, picking up different neuraminidase subtypes. By using both host and neuraminidase subtype as discrete traits, it was also shown that reassortment was preferentially occurring in Anseriformes species (ducks, geese, etc.).

To conclude, phylodynamic methods using viral sequence data with time, space and species metadata reveal complex transmission patterns and can be used to understand, track, model and ultimately inform disease control measures.

#### 2.4.2. Parallel Evolution and the Emergence of Highly-Pathogenic Avian Influenza A Viruses, by Marina Escalera-Zamudio

**Figure 8:**
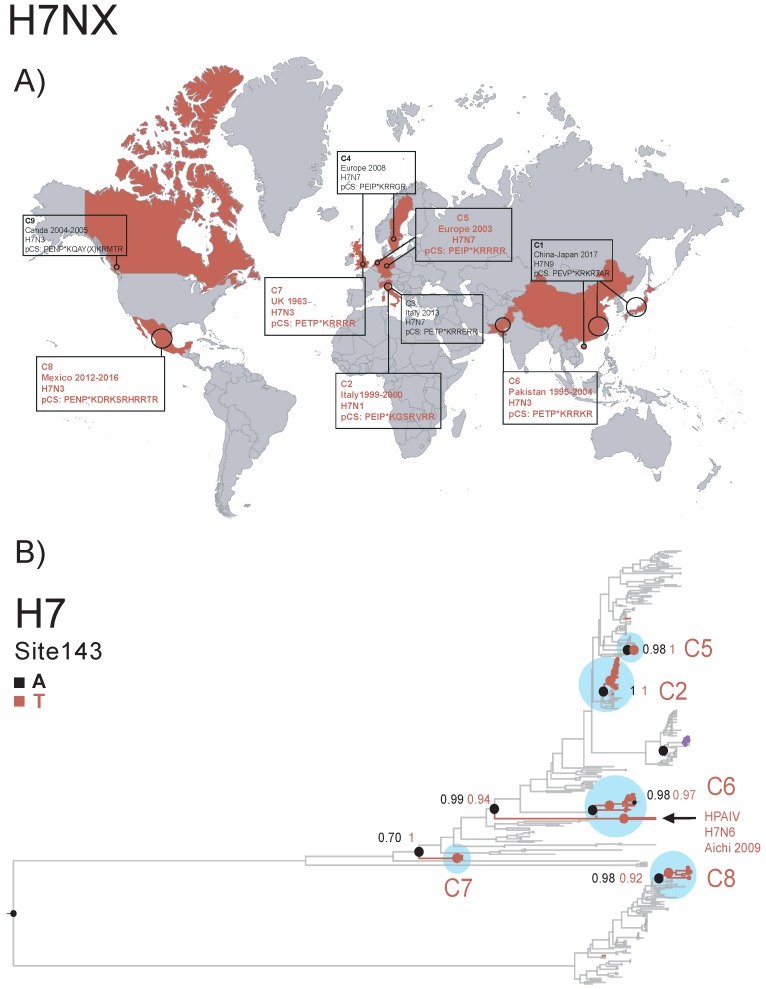
(**A**) Geographical occurrence of historic Highly-Pathogenic (HP) outbreaks for the H7NX viruses. Countries of emergence are highlighted in red. Year of circulation, virus subtype and consensus sequence for the polybasic Cleavage Site (pCS) within the Hemagglutinin (HA) protein are indicated for the selected outbreaks used in this work (C1-C9). Each outbreak corresponds to a distinct genotype, defined as well- supported clusters within all viral genome segment trees (data not shown). (**B**) MCCtree for the HA protein with reconstruction of ancestral states for site 143, as mutation A143T was found to be evolving under parallel evolution and to be associated with the HP phenotype, occurring in 4/9 of the HP clusters analysed. This mutation is a non-conservative amino acid change located within an antigenic pocket site. Branches within the trees are coloured according to the corresponding amino acid states in nodes (tip states not shown). Ancestral nodes preceding the emergence of a mutation associated with the HP lineages are represented with coloured circles. The probabilities of a given amino acid state occurring within ancestral/descending nodes are indicated. The HP clusters of interest are highlighted with blue circles. Mutations strongly associated with an HP phenotype may function as an early detection system for transitional virulence stages.

Avian Influenza A Viruses (AIVs) circulate among wild and domestic bird populations worldwide. While some strains only cause mild to asymptomatic infections, known as Low Pathogenicity avian influenza viruses (LP), High Pathogenicity avian influenza viruses (HP) can have an extremely high mortality rate in both domestic and wild bird populations, leading to huge economic loses (Figure 8A) [61]. Thus, surveillance of AIVs is crucial for early detection of outbreaks. Although virulence is a polygenic trait, molecular determinants of virulence have been well characterised for AIVs, such as a polybasic proteolytic cleavage site within the hemagglutinin protein, which enables a systemic viral spread within the host [62]. We hypothesise that the parallel evolution of HP lineages from LP ancestors may have been facilitated by permissive or compensatory secondary mutations occurring anywhere in the viral genome, preceding or following the appearance of a polybasic proteolytic cleavage site. We used a comparative phylogenetic and structural approach to detect shared mutations evolving under positive selection across the whole genome of HP AIVs of the H7NX and H5NX subtypes and developed a model that statistically assesses genotype-phenotype associations. We present cumulative evolutionary and structural evidence that supports the association between parallel mutations and the evolution of the HP phenotype. Parallel mutations occur frequently among HP lineages of the same viral subtype (Figure 8B). Many of the mutations have been previously determined to increase viral fitness in terms of their biological properties, whilst most of these are ranked as stabilising to protein structure, supporting that these are rather permissive/compensatory. The mutational panel provided here may function as an early detection system for transitional virulence stages. Circulating AIVs that do not have a polybasic cleavage site yet, but show all or some of the amino acid changes ranked, should remain under surveillance.

#### 2.4.3. Evolutionary Origins of Epidemic Potential among Human RNA Viruses, by Lu Lu

For a virus to have epidemic potential in human populations, an infected individual must be capable of transmitting the infection to other individuals. However, for the majority of human RNA virus species, human infections are acquired only from non-human reservoirs. The evolution of human transmissibility is poorly understood. Through parallel analyses of 1755 RNA viruses, we identified at least 90 nodes across 39 genus-level phylogenies associated with transitions involving the gain of human infectivity and/or transmissibility. Human-infective and human-transmissible viruses evolve independently, and at least 73% of human-transmissible RNA virus lineages emerged directly from non-human virus lineages in diverse mammal or bird taxa. Negative sense single-stranded RNA virus lineages generate a higher proportion of strictly zoonotic viruses. Our analysis demonstrates that RNA viruses from mammal/bird lineages not currently known to be infective to humans are a likely source of future epidemics in human populations, a public health threat recently designated “Disease X”.

## 3. Poster Session

Another important facet of this year’s annual EVBC meeting was the poster session on Thursday evening. The standard of the research presented was extremely high and, combined with a networking event in the Glasgow University Union, provided plenty of opportunity to meet the presenters. The relaxed atmosphere was instrumental to promoting discussions and developing new interactions between attendees. The list of poster presenters and titles can be found online (http://evbc.uni-jena.de/events/3rd-evbc-meeting).

## 4. Conclusions

The Third Annual Meeting of the European Virus Bioinformatics Center brought together scientists in the field with expertise in different disciplines for scientific exchange and provided the opportunity for discussing ongoing and new collaborations. The meeting attracted new researchers to virus bioinformatics, which was reflected by several first-time attendees. The presentations strongly underlined the interdisciplinary “virology meets bioinformatics” character of the meeting. We enjoyed lively discussions after the speakers’ presentations, in the breaks, during the poster session and at the social events.

We hope that speakers summaries provided in this report will give an interesting insight into the field of virus bioinformatics and will encourage interested researchers to join us at the Fourth Annual Meeting of the EVBC to be held in Switzerland in 2020. For more information, do not hesitate to contact us via evbc@uni-jena.de.

## Figures and Tables

**Table 1:** History of the Annual Meeting of the European Virus Bioinformatics Center(EVBC).

Date	Location	# of Participants	Key outcomes
6–8 March 2017	Friedrich Schiller University Jena, Germany	~100	Founding of the Center; Discussion of the role of EVBC; Election of the first Board of Directors; Insights into EU policy and funding opportunities.
9–10 April 2018	Utrecht University, Netherlands	~120	Extending of the EVBC network to include America and Asia; Discussion and design of joint projects; Insights on first applied European fund among EVBC members [3].
28–29 March 2019	University of Glasgow, United Kingdom	~110	Inclusion of contributed talks in themed sections in the scientific programme; Establishment of travel, poster and best contributed talk awards for junior scientists; Need for greater coordination and communication within the European virology community.

## References

[1] Hufsky F., Ibrahim B., Beer M., Deng L., Le Mercier P., McMahon D.P., Palmarini M., Thiel V., Marz M. (2018). Virologists—Heroes need weapons. PLoS Pathog..

[2] Ibrahim B., McMahon D.P., Hufsky F., Beer M., Deng L., Le Mercier P., Palmarini M., Thiel V., Marz M. (2018). A new era of virus bioinformatics. Virus Res..

[3] Ibrahim B., Arkhipova K., Andeweg A., Posada-Céspedes S., Enault F., Gruber A., Koonin E., Kupczok A., Lemey P., McHardy A. (2018). Bioinformatics Meets Virology: The European Virus Bioinformatics Center’s Second Annual Meeting. Viruses.

[4] V’kovski P., Gerber M., Kelly J., Pfaender S., Ebert N., Lagache S.B., Simillion C., Portmann J., Stalder H., Gaschen V. (2019). Determination of host proteins composing the microenvironment of coronavirus replicase complexes by proximity-labeling. eLife.

[5] Mayr F.B., Yende S., Angus D.C. (2014). Epidemiology of severe sepsis. Virulence.

[6] Singer M., Deutschman C.S., Seymour C.W., Shankar-Hari M., Annane D., Bauer M., Bellomo R., Bernard G.R., Chiche J.D., Coopersmith C.M. (2016). The Third International Consensus Definitions for Sepsis and Septic Shock (Sepsis-3). JAMA.

[7] Iuliano A.D., Roguski K.M., Chang H.H., Muscatello D.J., Palekar R., Tempia S., Cohen C., Gran J.M., Schanzer D., Cowling B.J. (2018). Estimates of global seasonal influenza-associated respiratory mortality: A modelling study. Lancet (London, England).

[8] Papanicolaou G.A. (2013). Severe influenza and S. aureus pneumonia: For whom the bell tolls?. Virulence.

[9] Klemm C., Bruchhagen C., van Krüchten A., Niemann S., Löffler B., Peters G., Ludwig S., Ehrhardt C. (2017). Mitogen-activated protein kinases (MAPKs) regulate IL-6 over-production during concomitant influenza virus and *Staphylococcus aureus* infection. Sci. Rep..

[10] van Krüchten A., Wilden J.J., Niemann S., Peters G., Löffler B., Ludwig S., Ehrhardt C. (2018). *Staphylococcus aureus* triggers a shift from influenza virus-induced apoptosis to necrotic cell death. FASEB J..

[11] Russell A.B., Trapnell C., Bloom J.D. (2018). Extreme heterogeneity of influenza virus infection in single cells. eLife.

[12] Steuerman Y., Cohen M., Peshes-Yaloz N., Valadarsky L., Cohn O., David E., Frishberg A., Mayo L., Bacharach E., Amit I. (2018). Dissection of Influenza Infection In Vivo by Single-Cell RNA Sequencing. Cell Syst..

[13] Jonsdottir H.R., Dijkman R. (2015). Characterization of Human Coronaviruses on Well-Differentiated Human Airway Epithelial Cell Cultures. Coronaviruses.

[14] James C.E., Davies E.V., Fothergill J.L., Walshaw M.J., Beale C.M., Brockhurst M.A., Winstanley C. (2015). Lytic activity by temperate phages of *Pseudomonas aeruginosa* in long-term cystic fibrosis chronic lung infections. ISME J..

[15] Shan J., Ramachandran A., Thanki A.M., Vukusic F.B.I., Barylski J., Clokie M.R.J. (2018). Bacteriophages are more virulent to bacteria with human cells than they are in bacterial culture; insights from HT-29 cells. Sci. Rep..

[16] Nale J.Y., Redgwell T.A., Millard A., Clokie M.R.J. (2018). Efficacy of an Optimised Bacteriophage Cocktail to Clear *Clostridium difficile* in a Batch Fermentation Model. Antibiotics.

[17] Nale J.Y., Spencer J., Hargreaves K.R., Buckley A.M., Trzepiński P., Douce G.R., Clokie M.R.J. (2016). Bacteriophage Combinations Significantly Reduce *Clostridium difficile* Growth In Vitro and Proliferation In Vivo. Antimicrob. Agents Chemother..

[18] Hendrix R.W., Smith M.C., Burns R.N., Ford M.E., Hatfull G.F. (1999). Evolutionary relationships among diverse bacteriophages and prophages: All the world’s a phage. Proc. Natl. Acad. Sci. USA.

[19] Sanjuán R., Nebot M.R., Chirico N., Mansky L.M., Belshaw R. (2010). Viral mutation rates. J. Virol..

[20] Breitbart M., Salamon P., Andresen B., Mahaffy J.M., Segall A.M., Mead D., Azam F., Rohwer F. (2002). Genomic analysis of uncultured marine viral communities. Proc. Natl. Acad. Sci. USA.

[21] Roux S., Brum J.R., Dutilh B.E., Sunagawa S., Duhaime M.B., Loy A., Poulos B.T., Solonenko N., Lara E., Poulain J. (2016). Ecogenomics and potential biogeochemical impacts of globally abundant ocean viruses. Nature.

[22] Breitbart M., Rohwer F. (2005). Here a virus, there a virus, everywhere the same virus?. Trends Microbiol..

[23] Dutilh B.E., Cassman N., McNair K., Sanchez S.E., Silva G.G.Z., Boling L., Barr J.J., Speth D.R., Seguritan V., Aziz R.K. (2014). A highly abundant bacteriophage discovered in the unknown sequences of human faecal metagenomes. Nat. Commun..

[24] Stern A., Mick E., Tirosh I., Sagy O., Sorek R. (2012). CRISPR targeting reveals a reservoir of common phages associated with the human gut microbiome. Genome Res..

[25] Manrique P., Bolduc B., Walk S.T., van der Oost J., de Vos W.M., Young M.J. (2016). Healthy human gut phageome. Proc. Natl. Acad. Sci. USA.

[26] Mahmoudabadi G., Phillips R. (2018). A comprehensive and quantitative exploration of thousands of viral genomes. eLife.

[27] Kang H.S., McNair K., Cuevas D., Bailey B., Segall A., Edwards R.A. (2017). Prophage genomics reveals patterns in phage genome organization and replication. bioRxiv.

[28] Miller E.S., Kutter E., Mosig G., Arisaka F., Kunisawa T., Ruger W. (2003). Bacteriophage T4 Genome. Microbiol. Mol. Biol. Rev..

[29] Brum J.R., Schenck R.O., Sullivan M.B. (2013). Global morphological analysis of marine viruses shows minimal regional variation and dominance of non-tailed viruses. ISME J..

[30] Mavrich T.N., Hatfull G.F. (2017). Bacteriophage evolution differs by host, lifestyle and genome. Nat. Microbiol..

[31] Kupczok A., Neve H., Huang K.D., Hoeppner M.P., Heller K.J., Franz C.M.A.P., Dagan T. (2018). Rates of Mutation and Recombination in Siphoviridae Phage Genome Evolution over Three Decades. Mol. Biol. Evol..

[32] Simmonds P., Aiewsakun P., Katzourakis A. (2018). Prisoners of war — host adaptation and its constraints on virus evolution. Nat. Rev. Microbiol..

[33] Edwards R., Vega A., Norman H., Ohaeri M.C., Levi K., Dinsdale E., Cinek O., Aziz R., McNair K., Barr J. (2019). Global phylogeography and ancient evolution of the widespread human gut virus crAssphage. bioRxiv.

[34] Mokili J.L., Rohwer F., Dutilh B.E. (2012). Metagenomics and future perspectives in virus discovery. Curr. Opin. Virol..

[35] Symonds E.M., Breitbart M. (2014). Affordable Enteric Virus Detection Techniques Are Needed to Support Changing Paradigms in Water Quality Management. Clean.

[36] Bibby K. (2013). Metagenomic identification of viral pathogens. Trends Biotechnol..

[37] Roux S., Adriaenssens E.M., Dutilh B.E., Koonin E.V., Kropinski A.M., Krupovic M., Kuhn J.H., Lavigne R., Brister J.R., Varsani A. (2018). Minimum Information about an Uncultivated Virus Genome (MIUViG). Nat. Biotechnol..

[38] Eren A.M., Esen Ö.C., Quince C., Vineis J.H., Morrison H.G., Sogin M.L., Delmont T.O. (2015). Anvi’o: An advanced analysis and visualization platform for ’omics data. PeerJ.

[39] Adriaenssens E., Farkas K., Harrison C., Jones D., Allison H.E., McCarthy A.J. (2018). Viromic analysis of wastewater input to a river catchment reveals a diverse assemblage of RNA viruses. bioRxiv.

[40] Simmonds P., Adams M.J., Benkő M., Breitbart M., Brister J.R., Carstens E.B., Davison A.J., Delwart E., Gorbalenya A.E., Harrach B. (2017). Consensus statement: Virus taxonomy in the age of metagenomics. Nat. Rev. Microbiol..

[41] Aiewsakun P., Adriaenssens E.M., Lavigne R., Kropinski A.M., Simmonds P. (2018). Evaluation of the genomic diversity of viruses infecting bacteria, archaea and eukaryotes using a common bioinformatic platform: Steps towards a unified taxonomy. J Gen Virol.

[42] Aiewsakun P., Simmonds P. (2018). The genomic underpinnings of eukaryotic virus taxonomy: Creating a sequence-based framework for family-level virus classification. Microbiome.

[43] Patterson Ross Z., Klunk J., Fornaciari G., Giuffra V., Duchêne S., Duggan A.T., Poinar D., Douglas M.W., Eden J.S., Holmes E.C. (2018). The paradox of HBV evolution as revealed from a 16th century mummy. PLoS Pathog..

[44] Krause-Kyora B., Susat J., Key F.M., Kühnert D., Bosse E., Immel A., Rinne C., Kornell S.C., Yepes D., Franzenburg S. (2018). Neolithic and medieval virus genomes reveal complex evolution of hepatitis B. eLife.

[45] Mühlemann B., Jones T.C., Damgaard P.d.B., Allentoft M.E., Shevnina I., Logvin A., Usmanova E., Panyushkina I.P., Boldgiv B., Bazartseren T. (2018). Ancient hepatitis B viruses from the Bronze Age to the Medieval period. Nature.

[46] Bar-Gal G.K., Kim M.J., Klein A., Shin D.H., Oh C.S., Kim J.W., Kim T.H., Kim S.B., Grant P.R., Pappo O. (2012). Tracing hepatitis B virus to the 16th century in a Korean mummy. Hepatology.

[47] Puustusmaa M., Abroi A. (2016). Conservation of the E8 CDS of the E8^*E*2^ protein among mammalian papillomaviruses. J. Gen. Virol..

[48] Puustusmaa M., Abroi A. (2019). cRegions—a tool for detecting conserved cis-elements in multiple sequence alignment of diverged coding sequences. PeerJ.

[49] Nicholson B.L., White K.A. (2014). Functional long-range RNA-RNA interactions in positive-strand RNA viruses. Nat. Rev. Microbiol..

[50] Madhugiri R., Karl N., Petersen D., Lamkiewicz K., Fricke M., Wend U., Scheuer R., Marz M., Ziebuhr J. (2018). Structural and functional conservation of cis-acting RNA elements in coronavirus 5’-terminal genome regions. Virology.

[51] Kuraku S., Zmasek C.M., Nishimura O., Katoh K. (2013). aLeaves facilitates on-demand exploration of metazoan gene family trees on MAFFT sequence alignment server with enhanced interactivity. Nucleic Acids Res..

[52] Will S., Reiche K., Hofacker I.L., Stadler P.F., Backofen R. (2007). Inferring noncoding RNA families and classes by means of genome-scale structure-based clustering. PLoS Comput. Biol..

[53] Pickett B.E., Sadat E.L., Zhang Y., Noronha J.M., Squires R.B., Hunt V., Liu M., Kumar S., Zaremba S., Gu Z. (2012). ViPR: An open bioinformatics database and analysis resource for virology research. Nucleic Acids Res..

[54] Fernández-Sanlés A., Ríos-Marco P., Romero-López C., Berzal-Herranz A. (2017). Functional Information Stored in the Conserved Structural RNA Domains of Flavivirus Genomes. Front. Microbiol..

[55] Filomatori C.V., Lodeiro M.F., Alvarez D.E., Samsa M.M., Pietrasanta L., Gamarnik A.V. (2006). A 5’ RNA element promotes dengue virus RNA synthesis on a circular genome. Genes Dev..

[56] Clyde K., Barrera J., Harris E. (2008). The capsid-coding region hairpin element (cHP) is a critical determinant of dengue virus and West Nile virus RNA synthesis. Virology.

[57] Kozak M. (1990). Downstream secondary structure facilitates recognition of initiator codons by eukaryotic ribosomes. Proc. Natl. Acad. Sci. USA.

[58] Global Consortium for H5N8 and Related Influenza Viruses (2016). Role for migratory wild birds in the global spread of avian influenza H5N8. Science.

[59] Lycett S., Tanya V.N., Hall M., King D., Mazeri S., Mioulet V., Knowles N., Wadsworth J., Bachanek-Bankowska K., Victor N.N. (2019). The evolution and phylodynamics of serotype A and SAT2 foot-and-mouth disease viruses in endemic regions of Africa. bioRxiv.

[60] Duchatel F., Bronsvoort M., Lycett S. (2018). Phylogeographic analysis and identification of factors impacting the diffusion of Foot-and-Mouth disease virus in Africa. bioRxiv.

[61] Dhingra M.S., Artois J., Dellicour S., Lemey P., Dauphin G., Von Dobschuetz S., Van Boeckel T.P., Castellan D.M., Morzaria S., Gilbert M. (2018). Geographical and Historical Patterns in the Emergences of Novel Highly Pathogenic Avian Influenza (HPAI) H5 and H7 Viruses in Poultry. Front. Vet. Sci..

[62] Abdelwhab E.M., Veits J., Ulrich R., Kasbohm E., Teifke J.P., Mettenleiter T.C. (2016). Composition of the Hemagglutinin Polybasic Proteolytic Cleavage Motif Mediates Variable Virulence of H7N7 Avian Influenza Viruses. Sci. Rep..

